# Targeting the bone marrow microenvironment: a novel therapeutic strategy for pre-B acute lymphoblastic leukemia

**DOI:** 10.18632/oncotarget.26720

**Published:** 2019-03-05

**Authors:** Rishi S. Kotecha, Laurence C. Cheung

**Affiliations:** Laurence C. Cheung: Telethon Kids Cancer Centre, Telethon Kids Institute, University of Western Australia, Perth, WA, Australia; School of Pharmacy and Biomedical Sciences, Curtin University, Perth, WA, Australia

**Keywords:** acute lymphoblastic leukemia, microenvironment, zoledronic acid, RANKL, bones

Acute lymphoblastic leukemia (ALL) is the most common form of cancer in children, accounting for approximately 25% of all childhood cancers. Treatment of pediatric ALL represents one of the success stories of modern medicine, with 5-year survival exceeding 90% [1]. Improved outcome can be attributed to risk-stratified therapy and the discovery of targeted agents. Despite such advances, a significant proportion of high-risk patients, such as infants with KMT2A gene rearrangements and children with BCR-ABL fusion, continue to have an inferior prognosis [2]. Although treatment protocols have increased the intensity of conventional chemotherapy to improve survival, chemotherapeutic drugs do not spare healthy normal cells and a plateau has been reached; recent years have seen little improvement in outcome due to an increase in toxic death and the recognition of increased morbidity in survivors due to the development of significant late effects.

Novel therapeutic strategies provide much promise to further combat childhood leukemia. Over the past decade, the striking rise of immunotherapies has revolutionized treatment for cancer. Immunotherapy has reached a groundbreaking milestone, with the FDA approving three immunotherapies for certain indications in pediatric and adult ALL in 2017 [3], with many others under continued development.

Another novel therapeutic strategy that remains primed for investigation is targeting of the tumor microenvironment. Recognition that the tumor microenvironment contributes to treatment failure or success has led to a paradigm shift [4]. While extensive research has identified that the microenvironment of solid tumors plays a key role in multiple stages of cancer progression, relapse, immune-escape and metastasis, the importance of the leukemia microenvironment has only recently been appreciated. Studies in acute myeloid leukemia have demonstrated that normalization of the bone marrow microenvironment reduces disease progression, validating the microenvironment as a new therapeutic target for patients with leukemia [5].

**Figure 1:**
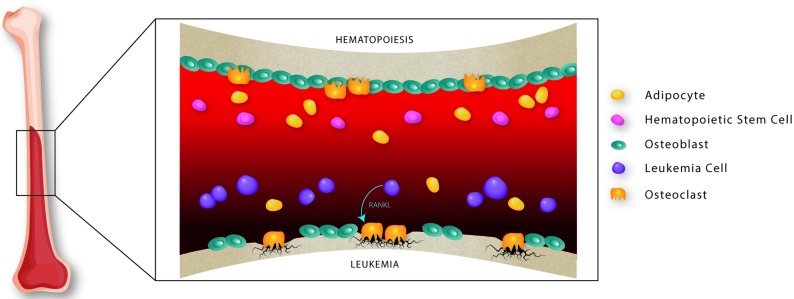
Schematic representation of normal *versus* the pre-B acute lymphoblastic leukemia bone marrow microenvironment In normal hematopoiesis, hematopoietic stem cells are surrounded by stromal cells in the microenvironment including osteoblasts, osteoclasts and adipocytes. In contrast, invasion of leukemia cells results in osteopenia as well as a reduction in osteoblast and adipocyte number. RANKL, produced by leukemia cells, is responsible for osteoclast-mediated bone resorption.

Changes within the bone marrow microenvironment have been described in patients with ALL (**Figure 1**). This includes lower serum markers of bone formation prior to initializing therapy, reduction in trabecular bone volume and thickness, as well as lower percentages of adipocytes, osteoblasts and osteoclasts [6–8]. However, the impact of ALL on hematopoiesis and the bone marrow microenvironment remains unclear. In a recent study, we characterized the bone marrow microenvironment in pre-B ALL using an immunocompetent BCR-ABL1^+^ model [9]. Leukemia development was shown to perturb B-lymphopoiesis and myelopoiesis, suggesting that leukemogenesis alters the activity of hematopoietic stem and progenitor cells. Furthermore, reduced numbers of osteoblasts and enhanced activity of osteoclasts contribute to the homeostatic imbalances of bone during leukemia development, recapitulating the clinical features of bone loss in children with ALL. A high level of RANKL secreted by leukemia cells was identified as being responsible for the osteoclast-mediated bone loss (**Figure 1**).

To test whether targeting of the bone marrow microenvironment reduced progression of pre-B ALL, the effect of an osteoclastic inhibitor, zoledronic acid, was investigated. Zoledronic acid has been used as an adjuvant therapy for treating advanced adult cancers with bone metastases and has been well tolerated when administered to children with ALL for treatment of osteonecrosis [10]. Treatment not only inhibited the activity of osteoclasts and restored bone loss but also hindered leukemia progression and prolonged survival.

In summary, our findings shed light on the mechanisms of leukemia-induced bone loss and provide evidence for targeting the pathological interplay within the leukemia microenvironment as a novel therapeutic strategy for patients with pre-B ALL. Further studies evaluating the efficacy of zoledronic acid in combination with standard chemotherapy for pre-B ALL are now warranted.
